# The efficiency of MSC‐based targeted AIE nanoparticles for gastric cancer diagnosis and treatment: An experimental study

**DOI:** 10.1002/btm2.10278

**Published:** 2021-12-24

**Authors:** Sushan Ouyang, Yi Zhang, Sheng Yao, Longbao Feng, Ping Li, Senlin Zhu

**Affiliations:** ^1^ Department of Gastroenterology and Hepatology The First Affiliated Hospital of Sun Yat‐sen University Guangzhou China; ^2^ Department of Hepatobiliary Surgery The Third Affiliated Hospital of Sun Yat‐sen University Guangzhou China; ^3^ Department of Gastroenterology The Second Affiliated Hospital of Zhejiang University School of Medicine Hangzhou China; ^4^ Beogene Biotech (Guangzhou) Co., Ltd. Guangzhou China

**Keywords:** cell‐penetrating peptide, gastric cancer, mesenchymal stem cells, paclitaxel, targeted, tetraphenylethylene

## Abstract

Mesenchymal stem cells (MSCs), due to their tumor tropism, are strongly recruited by various solid tumors and mobilized by inflammatory signals in the tumor microenvironment. However, effective cellular uptake is critical for MSC‐based drug delivery. In this study, we synthesized a spherical copolymer, polyethylenimine–poly(ε‐caprolactone), with aggregation‐induced emission (AIE) material and the anticancer drug, paclitaxel, coloaded onto its inner core. This was followed by the addition of a transactivator of transcription (TAT) peptide, a type of cell‐penetrating peptide, to modify the nanoparticles (NPs). Finally, the MSCs were employed to carry the TAT‐modified AIE‐NPs drug to the tumor sites and assist in simultaneous cancer diagnosis and targeted tumor therapy. In vitro, the TAT‐modified AIE‐NPs showed good biocompatibility, targeting, and stability in an aqueous solution besides high drug‐loading and encapsulation efficiency. In vitro, the AIE‐NPs exhibited a controllable release under a mildly acidic environment. The in vivo *and* in vitro studies showed high antitumor efficacy and low cytotoxicity of the AIE‐NP drug, whereas biodistribution confirmed the tumor tropism of MSCs. To summarize, the MSC‐based AIE‐NP drugs loaded with TAT possessed good biocompatibility and high antitumor efficacy via the enhanced NP‐drug uptake. In addition, the tumor tropism of MSCs provided selective drug uptake by the tumor cells and thus reduced the systemic side effects.

## 1 INTRODUCTION

Gastric cancer (GC) is a global health problem, ranking fifth in the incidence among all cancers (more than a million new cases during 2020), and fourth in cancer‐related mortality (estimated 769,000 deaths) worldwide.^1^ Although current molecular target agents, such as trastuzumab, improve the response rate to some extent,^2^ the 5‐year survival rate remains less than 10% for advanced‐stage patients.^3^ To reduce the incidence rate and prolong survival, efforts in developing less invasive early diagnosis tools and more effective treatment strategies are urgently needed.

Nanotechnology‐based drug delivery systems (DDS) can modify the size and shape of materials at the nanoscale (size range of 1–100 nm) to improve the cellular uptake and accessibility to the cancer cells by prolonging circulation time and increasing drug retention in tumor tissues, attributed to the impacts of enhanced permeability and retention.^4^ By modifying the surface of nanoparticles (NPs), nanocarriers can precisely deliver drugs to the cancer cells, release them in a controlled manner, and decrease off‐target toxicity. Liposome‐encapsulated doxorubicin (Doxil) and PEG–l‐asparaginase (Oncospar) are the two successful representatives used in clinics.^5^, ^6^ Many strategies such as stimuli‐responsive nanocarriers have been used to improve delivery efficiency^7^, ^8^; however, the rapid recognition and clearance of nanocarriers from the bloodstream by the reticuloendothelial system (RES) limit their usefulness as drug carriers.

**SCHEME 1:**
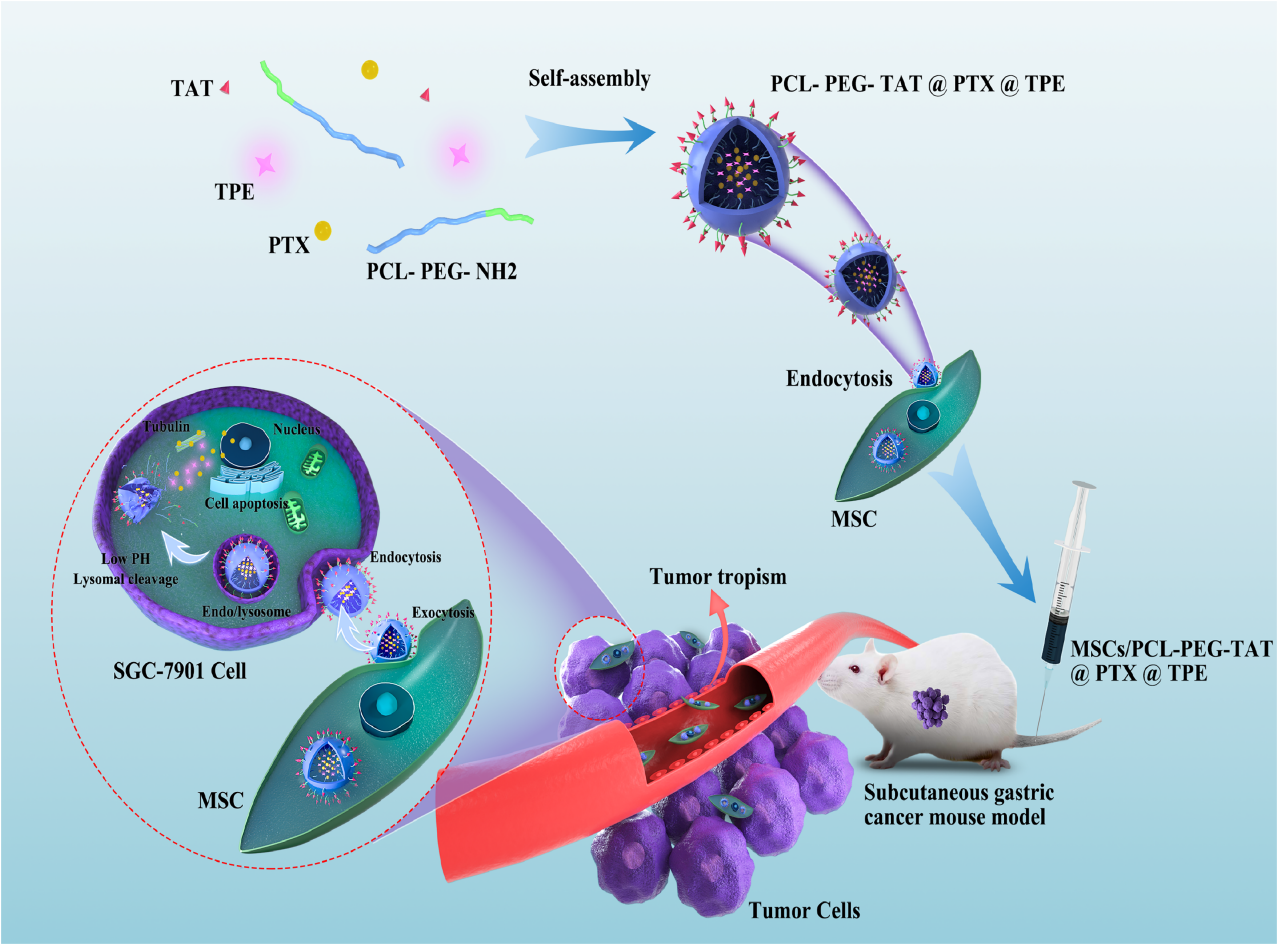
Schematic illustration of the MSC‐based NPs drug platform for targeted cancer therapy. The TAT peptide facilitates MSC and tumor cell uptake of the NP drugs. After the intravenous injection, the inherent tumor tropism of MSCs can target the tumor tissue and release the therapeutic cargo through exocytosis. Subsequently, the NP drug can be internalized by the tumor cells and released under the acidic environment of endo/lysosomes, inducing efficient apoptosis of the tumor cells. MSC, mesenchymal stem cells; NP, nanoparticle; TAT, transactivator of transcription

Previous research showed that mesenchymal stem cells (MSCs) are strongly recruited by various tumors and mobilized by inflammatory signals from the tumor microenvironment.^9^, ^10^, ^11^ The natural tumor tropism of MSCs and their ability to inoculate into cancer sites, along with their low immunogenicity, stability, and expandability, make them the ideal drug carrier for the delivery and improved bioavailability of anticancer agents.^12^, ^13^ The MSC‐based vehicles with drug‐loaded NPs provide an effective alternative to overcome the biodistribution limitations of NPs; the ability of MSCs to migrate within the tumor tissue protected the NPs from clearance by the body's immune system and enabled their entry into the tumor core.^8^ Studies have demonstrated that MSCs loaded with chemotherapeutic NPs homed to tumor sites and achieved cellular drug storage, which release the drug in a controlled manner in prostate, lung, and glioma animal models.^14^, ^15^ The imaging technique helps in the early diagnosis of cancers and tracking of real‐time drug release. Fluorescence imaging has been used widely in the biomedical field due to its rapid signal acquisition and high detection sensitivity^16^; however, the imaging performance of traditional fluorescent dyes incorporated in NPs at high loadings is limited by the aggregation‐caused quenching (ACQ) effect.^17^ The aggregation‐induced emission (AIE) phenomenon discovered by Tang et al. addressed the ACQ drawbacks.^18^ The AIE‐active materials have been used in bioimaging and drug delivery because they are nonluminescent in dilute solutions and become highly emissive when aggregated.^19^, ^20^ Tetraphenylethylene (TPE), an AIE fluorogen, possesses high biocompatibility and has been used extensively in bioimaging and drug delivery.^21^ This study used a novel MSC‐based platform with TPE loaded onto the copolymer polyethylenimine–poly(ε‐caprolactone) (PCL‐PEG), packeted with paclitaxel (PTX), and utilizing a cell‐penetrating peptide to modify the drug‐loaded NPs to enhance its cellular uptake (Scheme 1). We analyzed the physical and chemical characteristics of the drug‐loaded AIE‐NPs, biosafety of the synthesized NPs copolymers, and evaluated the uptake efficacy and drug release capacity of the bone marrow‐derived MSCs. Human GC (SGC‐7901 cell lines) xenograft nude mouse model was constructed to test the biodistribution and tumor tropism of MSCs. The efficient tumor targeting of MSCs enhanced the antitumor efficacy of NP drugs, while the fluorescent probes, TPE, enabled real‐time monitoring of drug localization and early diagnosis of cancer. Thus, the versatility of the MSC‐based platform may contribute to a new strategy for efficient cancer detection and therapy in the clinical setting.

## 2 MATERIALS AND METHODS

### 2.1 Materials

1‐Ethyl‐3‐(3‐dimethyl aminopropyl) carbodiimide hydrochloride (EDC), *N*‐hydroxysuccinimide (NHS), dimethyl sulfoxide (DMSO), poly(ε‐caprolactone)‐2000 (PCL‐2k), and TPE were acquired from Shanghai Macklin Biochemical Co., Ltd. Polyethylene glycol‐5000 (PEG‐5 k) was bought from JenKem Technology Co., Ltd. Chemically pure PTX was acquired from Shanghai Aladdin Bio‐Chem Technology Co., Ltd. Transactivator of transcription (TAT) was purchased from Sangon Biotech Co., Ltd. The purchase of tetrahydrofuran (THF) was made with Damao Chemical Reagent Factory. Methylene chloride was provided by Guangzhou Chemical Reagent Factory. Fetal bovine serum, trypsin, coupled with low glucose Dulbecco's modified Eagle's medium (L‐DMEM) were bought from Gibco. Cell counting kit‐8 (CCK‐8) was supplied by Dojindo Molecular Technologies, Inc. Becton, Dickinson, and Company served up Annexin V‐FITC apoptosis detection kit.

### 2.2 Methods

#### 2.2.1 Synthesis of PCL‐PEG

PCL‐PEG was synthesized by ring‐opening polymerization as described previously with slight modifications.^22^ First, the initiator benzyl alcohol was exploited for the synthesis of monohydroxy‐terminated poly(ε‐caprolactone) (PCL‐OH), with SnOct (0.1% moles of ε‐caprolactone) as a catalyst. Then, 4‐nitrophenyl chloroformate (NPC) (1.2 g, 3 mmol) and pyridine (0.4 ml, 5 mmol) were added to PCL‐OH (1.2 g, 0.6 mmol), dissolved in dichloromethane, and the reaction was carried out under the nitrogen atmosphere at room temperature for 24 h. Subsequently, we purified PCL‐NPC via precipitation in cold ether and dried it under vacuum. Afterward, THF solution (2 ml) containing PCL‐NPC (0.2 g, 0.1 mmol) and DMSO solution (5 mL) containing PEG‐5 k (1 g, 0.5 mmol) were mixed and agitated for 24 h at room temperature. Finally, ether was added into the above reaction solution to extract PCL‐PEG and vacuum dried.

#### 2.2.2 Synthesis of PCL‐PEG‐TAT

The TAT peptide stock solution was prepared with 12.28 mg of TAT dissolved in 200 μl of DMSO, followed by the addition of EDC and NHS to activate the TAT peptide for 4 h. Later, THF (10 ml) containing PCL‐PEG‐NH_2_ (20 mg) was dropped into the activated TAT peptide solution and maintained reaction for another 24 h. The PCL‐PEG‐TAT copolymer conjugates were then obtained by precipitating the mixture with cold ether. The solid product was washed three times and preserved at 4°C for subsequent use.

#### 2.2.3 Preparation of PCL‐PEG‐TAT/PTX/TPE

To prepare the PCL‐PEG‐TAT/PTX/TPE nanomicelles, the thin‐film hydration method was utilized.^23^ A small amount of PCL‐PEG‐TAT (30 mg), PTX (10 mg), and TPE (10 mg) were dissolved in dichloromethane (20 ml), and the mixture was placed in a rotary evaporator to obtain a dry film at 45°C under vacuum. Water (10 ml) was input into the dry film while the solution was vortexed and sonicated for 30 min. The obtained micelle was filtered through a millipore filter with a pore size of 0.8 μm to remove the unencapsulated PTX and TPE. The PTX‐loaded PCL‐PEG‐TAT/TPE micelles were conserved at 4°C for further experiments. The PCL‐PEG/PTX/TPE was prepared as mentioned in Section 2.2.2, but with PCL‐PEG instead of PCL‐PEG‐TAT.

### 2.3 Characterization

^1^H‐NMR of PCL‐PEG‐NH_2_, TPE, PTX, and PCL‐PEG/TPE/PTX was carried out on an Avance III‐500 spectrometer (Bruker Corporation). The MestReNova software (Masterlab Research) was used for spectrum analysis. The Fourier transform infrared (FT‐IR) spectra of PCL‐PEG‐NH_2_, TPE, PTX, and PCL‐PEG/TPE/PTX were detected on a VERTEX‐70 instrument (Bruker Corporation) at room temperature. Sample flakes were detected using an FT‐IR spectrometer at a wavenumber range of 4000–500 cm^−1^. We employed transmission electron microscope (TEM) H‐800 (Hitachi) to examine the morphologies of PCL‐PEG‐NH_2_, PCL‐PEG‐TAT, and PCL‐PEG‐TAT/TPE/PTX, their size distribution, and to take photographs. Particle size and distribution of PCL‐PEG‐NH_2_, PCL‐PEG‐TAT, and PCL‐PEG‐TAT/TPE/PTX in an aqueous solution were determined using a Nano Malvern instrument ZS90 (Malvern Panalytical) at room temperature. The fluorescence spectrum analysis of PCL‐PEG/TPE was done by preparing different concentrations with a gradient dilution ratio of 1 mg/ml, 800, 600, 400, 200, 100, 50, and 25 μg/ml (the TPE concentration was calculated). A Thermo Scientific MK3 microplate reader (Thermo Fisher Scientific) was used for fluorescence measurements, 365 and 475 nm were chosen as the excitation and emission wavelengths, respectively, and the slit width was 10 nm. The fluorescence spectra of PCL‐PEG/TPE were photographed under 365 nm ultraviolet light irradiation.

### 2.4 Encapsulation efficiency and loading capacity

A small amount (10 mg) of PCL‐PEG/TPE/PTX was deliquesced in DMSO and diluted to a final volume of 10 ml. Centrifugation (10,000 rpm for 10 min) was performed to obtain the supernatant. To quantify the released PTX, an ultraviolet spectrometer (UV‐2550; SHIMADZU) at 210 nm was employed. The LC% and EE% were calculated as follows (*n* = 3):

LC%=Amount ofPTXinNPs/NP'sweight×100%,

$$\mathrm{EE}\%=\left(\text{Amount of}\;\mathrm{PTX}\;\text{in}\;\mathrm{NPs}\right)/\left(\text{Total amount of}\;\mathrm{PTX}\;\text{added}\right)\times 100\%.$$

### 2.5 In vitro drug release

We used Zhang et al.'s method to evaluate the release properties of PTX from PCL‐PEG/TPE/PTX by performing all the experiments at 37°C, pH 5.7 in phosphate‐buffered saline (PBS).^24^ PCL‐PEG/TPE/PTX (10 mg) was resuspended in PBS (5 ml) with gentle shaking in the dark. At each time points (0, 0.25, 0.5, 2, 6, 12, 20, 30, 44, 56, 72, 86, and 98 h), the following procedure was repeated: 1 ml of solution was taken and centrifuged to collect the supernatant at 4200 rpm for 5 min in an ultraviolet spectrophotometer. Equal volumes of PBS and the supernatant were added to the residual sediment and were resuspended with gentle shaking. The PTX concentration in the samples at various time points was measured by an ultraviolet spectrophotometer as described in section.

### 2.6 Cell cytotoxicity and viability

The cell cytotoxicity of PCL‐PEG/PTX and PCL‐PEG‐TAT/PTX on SGC‐7901 cells and MSCs were evaluated by the CCK‐8 assay. The SGC‐7901 cells and MSCs were seeded into a 96‐well plate (5 × 10^3^ cells/well) separately and incubated overnight in a CO_2_ incubator. At the next step, the culture medium was removed and substituted by a complete medium comprising assorted PCL‐PEG/PTX and PCL‐PEG‐TAT/PTX concentrations (PTX concentration range 0–40 μg/ml), with five wells for each concentration. After 24‐h incubation, CCK‐8 solution (10 μl) was applied to each well and incubated for 1 h. A microplate reader measured the optical density (OD) at the wavelength of 450 nm. Cells without PTX exposure served as control. The cytotoxicity of blank PCL‐PEG‐TAT copolymer was evaluated similarly using the SGC‐7901 cells and MSCs with a copolymer concentration gradient from 1 to 320 μg/ml. Cell viability was computed by applying the formula below:

$$\text{Cell viability}\;\left(\%\right)=\left(\mathrm{OD}\;\text{of the experimental group}-\mathrm{OD}\;\text{of the blank group}\right)/\left(\mathrm{OD}\;\text{of the negative control group}-\mathrm{OD}\;\text{of the blank group}\right)\times 100\%.$$

### 2.7 Cell endocytosis study

The efficacy of TAT in mediating cellular uptake of PCL‐PEG/TPE was investigated. The MSCs with a density of 5 × 10^4^ cells per well were seeded in a 24‐well plate and incubated overnight to allow attachment at 37°C under 5% CO_2_. Afterward, the cells were exposed to a complete medium containing PCL‐PEG/TPE and PCL‐PEG‐TAT/TPE and incubated at four individual intervals (0.5, 2, 4, and 6 h). At the set time points, the cells in each group were washed with PBS to remove any free materials, digested with trypsin, and centrifugated (1000 rpm, 5 min). Subsequently, we collected the cells and resuspended them in 200 μl of PBS. The Accuri C6 flow cytometry (BD Biosciences) was used to detect the fluorescence intensity of all samples, further quantified with FlowJo 7.6.1 software (FlowJo LLC) (3 parallels per well).

### 2.8 Intracellular distribution

We further studied the intracellular distribution of PCL‐PEG‐TAT/TPE. The MSCs were exposed to 20 μg/ml PCL‐PEG‐TAT/TPE for 0.5, 2, 4, and 6 h. Then, MSCs were washed with PBS (2 ml) and stained with Lyso‐Tracker Green (75 nM) for 1 h. The cells were then fixed with 4% paraformaldehyde for 30 min, followed by a PBS wash. A confocal laser scanning microscopy (CLSM) (LSM880; Zeiss) was used for observing the intracellular distribution of PCL‐PEG‐TAT/TPE.

### 2.9 Intracellular drug retention in MSCs

The MSCs (density 5 × 10^4^ cells/cm^2^) were incubated with the PCL‐PEG/TPE/PTX or PCL‐PEG‐TAT/TPE/PTX of 20 μg/ml for 6 h, then washed three times with PBS to remove the free PCL‐PEG/TPE/PTX or PCL‐PEG‐TAT/TPE/PTX, and a fresh culture medium was added. After culturing for 0, 6, 24, 48, and 72 h, the MSCs were washed and followed by fixation with 4% paraformaldehyde for 30 min. Then, MSCs with PBS for three washes were treated with 0.5% Triton X‐100 for 10 min, rewashed three times with PBS, and then incubated with phalloidin in darkness for 30 min. Finally, the antifluorescent quenching sealant (10 μl) was added at 4°C and sealed lightly. The CLSM was used to observe the contents of the PCL‐PEG/TPE/PTX or PCL‐PEG‐TAT/TPE/PTX in the MSCs at the set time points.

### 2.10 In vitro migration assay

A Transwell assay was conducted to evaluate the effect of PCL‐PEG/TPE, PCL‐PEG/TPE/PTX, and PCL‐PEG‐TAT/TPE/PTX on the tumor‐homing capacity of the MSCs. The MSCs were treated with PCL‐PEG/TPE, PCL‐PEG/TPE/PTX, and PCL‐PEG‐TAT/TPE/PTX, respectively, for 24 h. Subsequently, the MSCs were trypsinized and centrifuged to remove the supernatant. The MSCs were resuspended with DMEM and seeded in the upper chamber at 2 × 10^4^ cells/well, and the SGC‐7901 cells were placed into the lower chamber at 2 × 10^4^ cells/well. The untreated MSCs served as a control. After seeding for 24 h, cells that failed to migrate through the pores were removed with cotton swabs. Later, methanol was used to fix the cells on the lower surface of the membrane and 1% crystal violet was used for staining for 20 min. Finally, we used an inverted fluorescence microscope to observe and count the migrated cells in three randomly selected microscopic fields (×400). The in vitro migration rate was calculated as:

$$\text{Migration rate}=\text{counts}\;\left(\text{sample group}\right)/\text{counts}\;\left(\text{control group}\right)\times 100\%.$$

### 2.11 In vitro antitumor effect of drug‐loaded MSCs

We conducted the Transwell assay to assess the antitumor efficiency of the drug‐loaded MSCs on the SGC‐7901 cells. A 24‐well (pore size 0.4 mm) plate was used for the SGC‐7901 cell seeding and culture for 24 h. The MSCs and the PCL‐PEG‐TAT/TPE/PTX‐loaded MSCs were added and cocultured with the SGC‐7901 cells (MSCs/SGC‐7901 ratio 1:10). The CCK‐8 assay was used to determine the SGC‐7901 cell viability using the supernatant removed after 1, 2, and 3 days of coculturing.

### 2.12 Apoptosis assay

The antiproliferation of MSCs loaded with PTX‐NPs on SGC‐7901 was also determined by the Transwell assay. The procedures were the same as in Section 2.11. The SGC‐7901 cells were treated with different formulations. After being processed for 24 h, cells were trypsinized, washed with PBS, resuspended in 200 μl of binding buffer, and dyed by Annexin V‐PE (5 μl) and 7‐AAD (5 μl) for 15 min. Cell apoptosis of each group was analyzed by the flow cytometer (Accuri C6; BD Biosciences).

### 2.13 Animal experiments

#### 2.13.1 Animal model

The animal research was approved by the Institutional Ethics Committee for Clinical Research and Animal Trials of the First Affiliated Hospital of Sun Yat‐sen University. BALB/c nude mice (female, 5–6 weeks old) purchased from Zhuhai BesTest Bio‐Tech Co., Ltd., were raised under specific‐pathogen‐free conditions. SGC‐7901 cells were dissociated with trypsin and resuspended with PBS. The xenograft tumor model was constructed by subcutaneously injecting 100 μl of the cell suspension containing 1.5 × 10^6^ SGC‐7901 cells into the armpit of the left forelimb.

#### 2.13.2 Tumor inhibition evaluation

When the tumor volume grew to approximately 100 mm^3^, the mice were randomized into six groups (*n* = 4). For cargo loading, MSCs in passages 3–6 were prepared and incubated with PCL‐PEG/TPE/PTX and PCL‐PEG‐TAT/TPE/PTX in L‐DMEM for 24 h at 37°C, respectively. Subsequently, 0.1 ml of PBS, MSCs, PTX, PCL‐PEG/TPE/PTX, MSCs‐PCL‐PEG/TPE/PTX, and MSCs‐PCL‐PEG‐TAT/TPE/PTX were intravenously administrated into the mice of each group. For agents containing PTX, the administration dosage was 1 mg PTX‐equiv./kg. The agents were injected every 3 days for a total treatment period of 21 days. The measurement of the body weight and the tumor size was taken every 3 days. The tumor volume was computed according to:

$$\text{Volume}=1/2\times \text{length}\times {\text{width}}^{2}.$$

Mice were euthanized after 21‐day treatment, and tumor tissues were dissected, photographed, and fixed with 4% formaldehyde. Hematoxylin and eosin (H&E) staining, terminal deoxynucleotidyl transferase dUTP nick end labeling (TUNEL), and immunohistochemical analysis of tumor tissues were performed.

### 2.14 Immunohistochemical assay

The antitumor effects of NPs‐PTX complex on tumor angiogenesis and tumor cell proliferation were gauged by platelet endothelial cell adhesion molecule (also named cluster of differentiation 31 [CD31]) and nuclear protein Ki67 immunohistochemical staining of tumor sections using the corresponding antibodies of CD31 and Ki67, respectively.

### 2.15 In vivo distribution study

For distribution evaluation, MSCs/PCL‐PEG‐TAT‐Cy5 (200 μl, 1.5 × 10^6^ MSCs in passage 3) was given intravenously. The time‐dependent biodistribution of MSCs‐PCL‐PEG‐TAT‐Cy5 and PCL‐PEG‐Cy5 in the mice were pictured by an IVIS Lumina imaging system (Caliper Life Sciences) at the intervals of 0.5, 1, 3, 5, and 8 h. After the 8‐h injection, all mice were euthanized, and the tumor tissues and vital organs (heart, liver, spleen, lungs, kidneys) were isolated and excised for the ex vivo fluorescence imaging. The wavelength of 640 nm was set as the excitation source, while the emission wavelength was the waveband of Cy5. PCL‐PEG‐Cy5 without MSCs loading was used as the control to determine the in vivo tumor tropism of MSCs.

### 2.16 In vivo biocompatibility evaluation

The toxicity of different formulations was also investigated in vivo. After 21 days of treatment, blood samples of each group were collected through retro‐orbital plexus into centrifuge tubes and rested at room temperature for 2 h. Afterward, centrifugation was used to separate the serum from the cells (3000 rpm, for 5 min). The upper serum was kept at −80°C for subsequent evaluation of biochemical parameters, including enzyme activity of alanine aminotransferase (ALT), aspartate aminotransferase (AST), lactate dehydrogenase (LDH), alkaline phosphatase (ALP), and values of albumin (ALB), creatinine (CREA), uric acid (UA), urea (UREA), and glucose (GLU). Euthanasia was used for the mice, and their vital organs, including the heart, liver, spleen, lungs, and kidneys, were dissected for tissue analysis by the H&E staining.

### 2.17 Statistical analysis

Results are represented as mean ± *SD*. For each group, at least three replicates were set, and two independent experiments were conducted. The two‐tailed Student's *t*‐test assuming equal variance was used for calculating significance between groups, and *p* < 0.05 was considered statistically significant.

## 3 RESULTS AND DISCUSSION

### 3.1 Synthesis and characterization of PCL‐PEG/PTX/TPE

**FIGURE 1:**
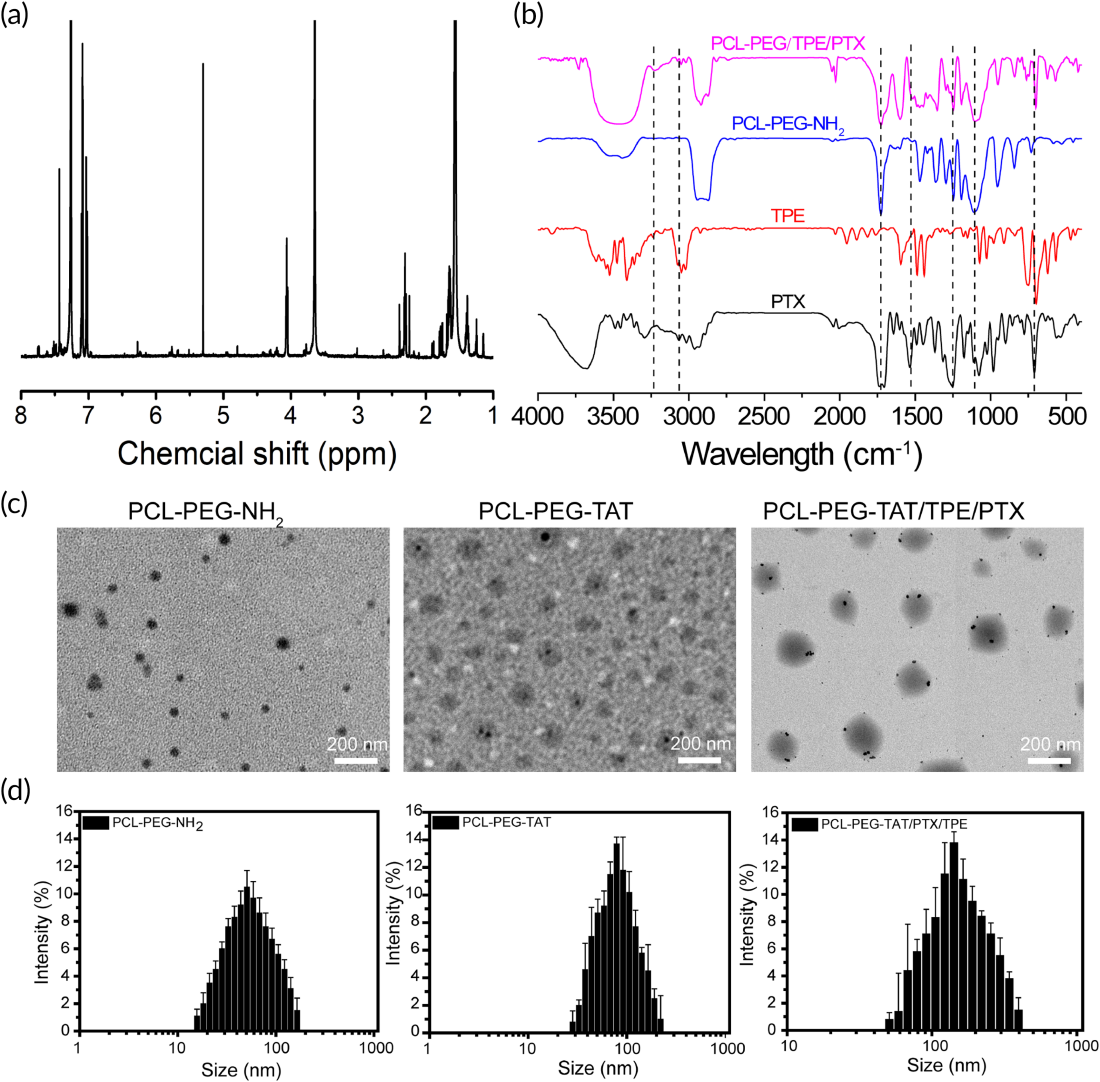
(a) ^1^H‐NMR spectrum of PCL‐PEG/TPE/PTX. (b) FT‐IR spectrum of PCL‐PEG/TPE/PTX, PCL‐PEG‐NH_2_, TPE, and PTX. (c) TEM images. (d) DLS of PCL‐PEG‐NH_2_, PCL‐PEG‐TAT, and PCL‐PEG‐TAT/TPE/PTX. Scale bars = 200 nm. DLS, dynamic light scattering; FT‐IR, Fourier transform infrared; PCL‐PEG, polyethylenimine–poly(ε‐caprolactone); PTX, paclitaxel; TAT, transactivator of transcription; TEM, transmission electron microscope; TPE, tetraphenylethylene

After being synthesized, the PCL‐PEG‐NH_2_ was characterized by ^1^H‐NMR spectroscopy (Figure S1). Methylene (CH_2_) in PEG was observed around 3.50 ppm. The methylene proton single peak and the multipeaks of the –O–CO–CH_2_–CH_2_–CH_2_–CH_2_–CH_2_– subunit in PCL were 2.15 and 1.49–1.69 ppm, respectively, indicating successful synthesis of PCL‐PEG‐NH_2_. TPE and PTX were loaded onto PCL‐PEG by self‐assembly later. The ^1^H‐NMR spectrum of PCL‐PEG/TPE/PTX (Figure 1a) shows proton peaks at 6.7–7.2 ppm representing the benzene rings of TPE, and the peak around 4.3 ppm representing PTX; the characteristic proton peaks of PCL‐PEG‐NH_2_ were also observed, suggesting the successful formation of the PCL‐PEG/TPE/PTX complex.^25^, ^26^, ^27^ Sharp and intense bands of the PCL‐PEG‐NH_2_ were shown at 1732 cm^−1^ in the FT‐IR spectrum, which were caused by the stretching of carboxylic ester (C=O) groups in PCL. The bands at 1115 and 1246 cm^−1^ were attributed to stretching and vibration of the C–O–C bond of the repeat units of –O–CH_2_–CH_2_ and its corresponding COO– bond in PEG. The successful generation of the PCL‐PEG‐NH_2_ was confirmed by these characteristic peaks. The absorption bands at 1545 and 708 cm^−1^ were attributed to the skeleton vibrations of the benzene ring of PTX, while the peak at 3030 cm^−1^ was the C–H bending vibrations of TPE. These results confirm the successful synthesis of PCL‐PEG/TPE/PTX (Figures 1b and S2).^28^, ^29^, ^30^ The TEM photographs show that PCL‐PEG‐NH_2_, PCL‐PEG‐TAT, and PCL‐PEG‐TAT/TPE/PTX are generally spherical and regular‐sized, with the mean size of 51.3, 94.4, and 154.2 nm, respectively (Figure 1c). Meanwhile, dynamic light scattering was adopted to measure the size of the blank PCL‐PEG‐NH_2_, and the average diameter is 65.6 nm with a polydispersity index (PdI) of 0.18. After being modified with TAT peptide, the mean size increased to 118.3 nm with a PdI of 0.10. When TPE and PTX were wrapped in PCL‐PEG‐TAT, the mean size was about 148 nm and the PdI was 0.14 (Figure 1d). The smaller size observed from the TEM photographs was presumably due to dehydration of the NP copolymers. The morphology, size, and Pdl indicate that the PTX‐loaded copolymers had good aqueous dispersibility, stability, and uniformity. The mean diameter of such NPs was less than 200 nm, making them easier to be engulfed by MSCs.

### 3.2 AIE behavior

**FIGURE 2:**
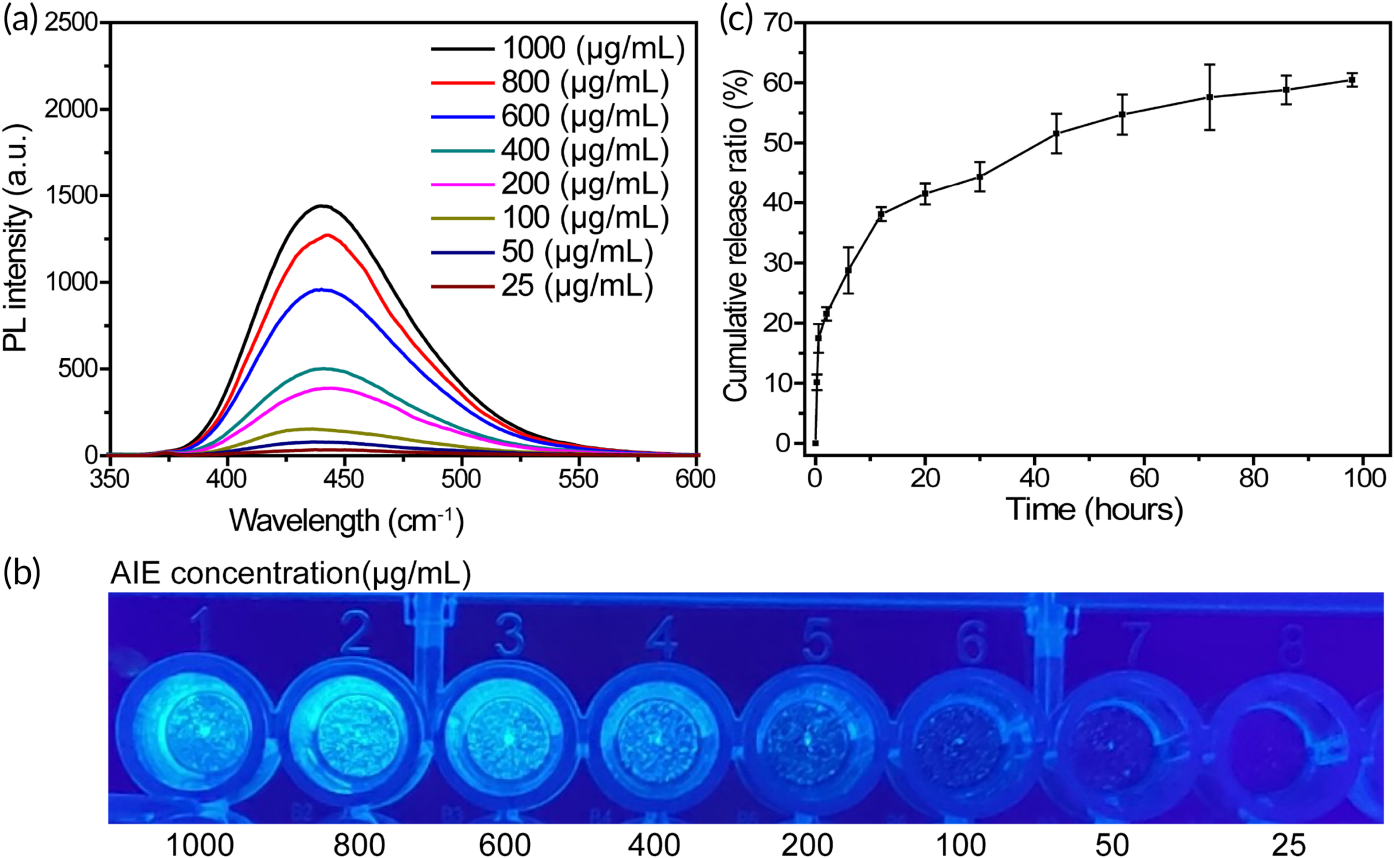
(a) Fluorescence spectrum of TPE with different concentrations. (b) Emission spectrum of TPE with different concentrations at an excitation wavelength of 365 nm. Characteristic peaks indicated with dash lines. (c) In vitro cumulative release curve of PTX from PCL‐PEG /TPE/PTX over time. PCL‐PEG, polyethylenimine–poly(ε‐caprolactone); PTX, paclitaxel; TPE, tetraphenylethylene

The molecules of TPE in a dissolved state vibrate and rotate freely. The molecules absorb energy and transfer the energy through a molecule movement, resulting in weak light emissions. Moreover, in an aggregation state, the inside movements of the TPE molecules are restricted, inducing a light emission. In this study, we prepared PCL‐PEG/TPE solutions with different concentrations, which were almost nonemissive at the condensation of 25 μg/ml, but the fluorescence intensity enhanced with increased concentrations. At the condensation of 1000 μg/ml, a strong aggregated‐state emission was observed (Figure 2a). At a high concentration, the aggregated molecules were visible to the naked eye, and a strong luminescence could be observed at a wavelength of 365 nm (Figure 2b). The luminescence intensity at 442 nm grew with increasing concentrations (Figure S3, linear equation *Y* = 1.487*X* + 10.567), consistent with the typical AIE observations.^31^

### 3.3 Drug‐loading and drug release capacity

The OD of PCL‐PEG/TPE/PTX (100 μg/ml) was calculated to be 1.15, according to the standard curve equation of PTX,

Y=0.0835X+0.0113,

with a correlation coefficient of *R* ^2^ = 0.999. The LC and EE were 13.64% and 63.82%, respectively. The internal hydrophobic structure of PCL‐PEG contributes enough space to load PTX, which helps achieve high drug‐loading efficacy. Figure 2c shows the release curve of free PTX and the cumulative release curve of PTX from PCL‐PEG/TPE/PTX in PBS (pH 5.7). The free PTX was released at a zero‐order release phase, whereas the PTX from NP complex was released at a relatively fast rate for the first 20 h; a cumulative release of 41.5%. Then, the release rate gradually slowed down, and the cumulative release at 98 h was 60.49%. The initial rapid release of PTX from the NPs at pH 5.7 was partially due to the rapid degradation of the PCL‐PEG copolymers under acidic conditions and partially attributed to the carboxylic groups' protonation in the PCL‐PEG copolymers, which weakened the electrostatic interactions between the cationic PTX and the anionic polymer carrier.^32^, ^33^ Intensely hydrophobic interaction between PTX and PCL led to the slow‐release phase.^34^ The rapid release under a lower pH condition favors the release of PTX from NPs in the acidic environment of the endo/lysosomes in the tumor cells, thus exerting a therapeutic function.

### 3.4 In vitro cell cytotoxicity analysis

**FIGURE 3:**
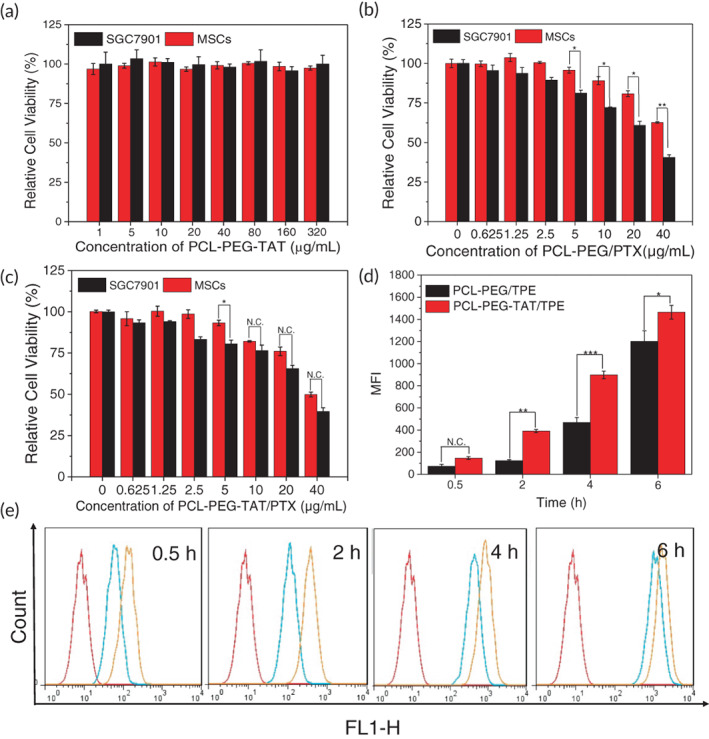
(a) Cell viability of SGC‐7901 cells and MSCs exposed to various concentrations of PCL‐PEG‐TAT. Cytotoxicity of different concentrations of PCL‐PEG/PTX (b) and PCL‐PEG‐TAT/PTX (c) in SGC‐7901 cells and MSCs. Mean fluorescence intensity (MFI) (d) and flow cytometry profiles (e) of MSCs incubated with PCL‐PEG/TPE and PCL‐PEG‐TAT/TPE. Bars represent the mean ± standard deviation (*p < 0.05, **p < 0.01, ***p < 0.001). MSC, mesenchymal stem cells; PCL‐PEG, polyethylenimine–poly(ε‐caprolactone); PTX, paclitaxel; TAT, transactivator of transcription; TPE, tetraphenylethylene

The SGC‐7901 and MSCs viability after incubation with various condensations of PCL‐PEG‐TAT were gauged by the CCK‐8 assay (Figure 3a). A relatively high cell viability was retained after incubation with PCL‐PEG‐TAT (concentration 1–320 μg/ml) for 24 h. Albeit a concentration of 320 μg/ml, a high survival rate of >90% of both types of cells was maintained. Thus, it can be said that the TAT modified PCL‐PEG copolymers possessed excellent biocompatibility, low cell cytotoxicity, and were safe as a drug carrier for tumor therapy. We further evaluated the cytotoxicity of PTX‐loaded PCL‐PEG and PCL‐PEG‐TAT on SGC‐7901 and MSCs with CCK‐8. The relative cell viability of both cells was concentration‐dependent (Figure 3). The survival rate of SGC‐7901 cells and MSCs decreased with increasing PCL‐PEG/PTX concentration. At the concentration of 40 μg/ml, the survival rate of SGC‐7901 and MSCs was 30% and 52%, respectively (Figure 3b), indicating inhibition of the two cells at that concentration. After the conjugation of the TAT peptide to PCL‐PEG/PTX, the cell viability decreased further compared to PCL‐PEG/PTX at the same concentration (Figure 3c), probably due to the increased drug uptake enhanced by the TAT peptide.^35^ We set the PTX concentration at 20 μg/ml for in vivo experiments according to the above results. The effect of bare MSCs or MSCs/PCL‐PEG‐TAT/TPE/PTX on SGC‐7901 cell viability was also determined via the CCK‐8 assay (Figure 5a). Bare MSCs did not affect the tumor cell viability or tumor growth. In contrast, when SGC‐7901 cells were cocultured with drug‐loaded NPs‐MSCs, the tumor cell viability was inhibited, with 70% on Day 1 and 40% on Day 3. The live/dead assay was carried out to examine the antitumor activity of the cells; the staining solution was added into the cocultured SGC‐7901 cells and MSCs (after 3 days of coculture) and incubated for 5 min at room temperature—the green and red represented the live cells and dead cells, respectively. In addition, an Annexin V‐PE/7‐AAD kit (5 μl of Annexin V‐PE and 5 μl of 7‐AAD, stained for 15 min) was employed to stain the SGC‐7901 cells for apoptosis analysis via a flow cytometry after 3 days of incubation with different formulations. It was observed that almost all SGC‐7901 cells were still living (green color) after coculturing with bare MSCs. Extensive dead cells (red color) were observed in the SGC‐7901 cells cocultured with MSCs carrying NP drugs (Figure S4). These results indicated that a considerable amount of NP drugs could be incorporated by MSCs, and PTX loaded with an NP copolymer decreased toxicity of MSCs, which is vital for MSCs as a drug vehicle. It also demonstrated that NP drugs could be excreted sustainably by MSCs at the tumor sites. Together, the results indicated the biosafety of MSCs as a cell‐based drug carrier. With inherent tumor tropism, efficient uptake, and exocytosis of therapeutic cargo at tumor sites, MSCs primed PCL‐PEG‐TAT/TPE/PTX could achieve a significant and prolonged antitumor activity.

### 3.5 TAT transduction efficiency

To confirm the intracellular delivery efficacy of TAT‐modified NPs, we performed flow cytometry analysis to quantify the blue fluorescence intensity in MSCs. For the MSCs cells incubated with an equivalent concentration of PCL‐PEG/TPE and PCL‐PEG‐TAT/TPE for the same time, it was evident that the mean fluorescence intensity of MSCs incubated with TAT‐loaded PCL‐PEG/TPE was much stronger than the MSCs incubated with PCL‐PEG/TPE over time (Figure 3d, *p* < 0.05). A significant gap was seen at the fourth hour (Figure 3e). The data validated that TAT modification remarkably enhanced cellular uptake of PCL‐PEG/TPE.

### 3.6 Drug retention in MSCs

**FIGURE 4:**
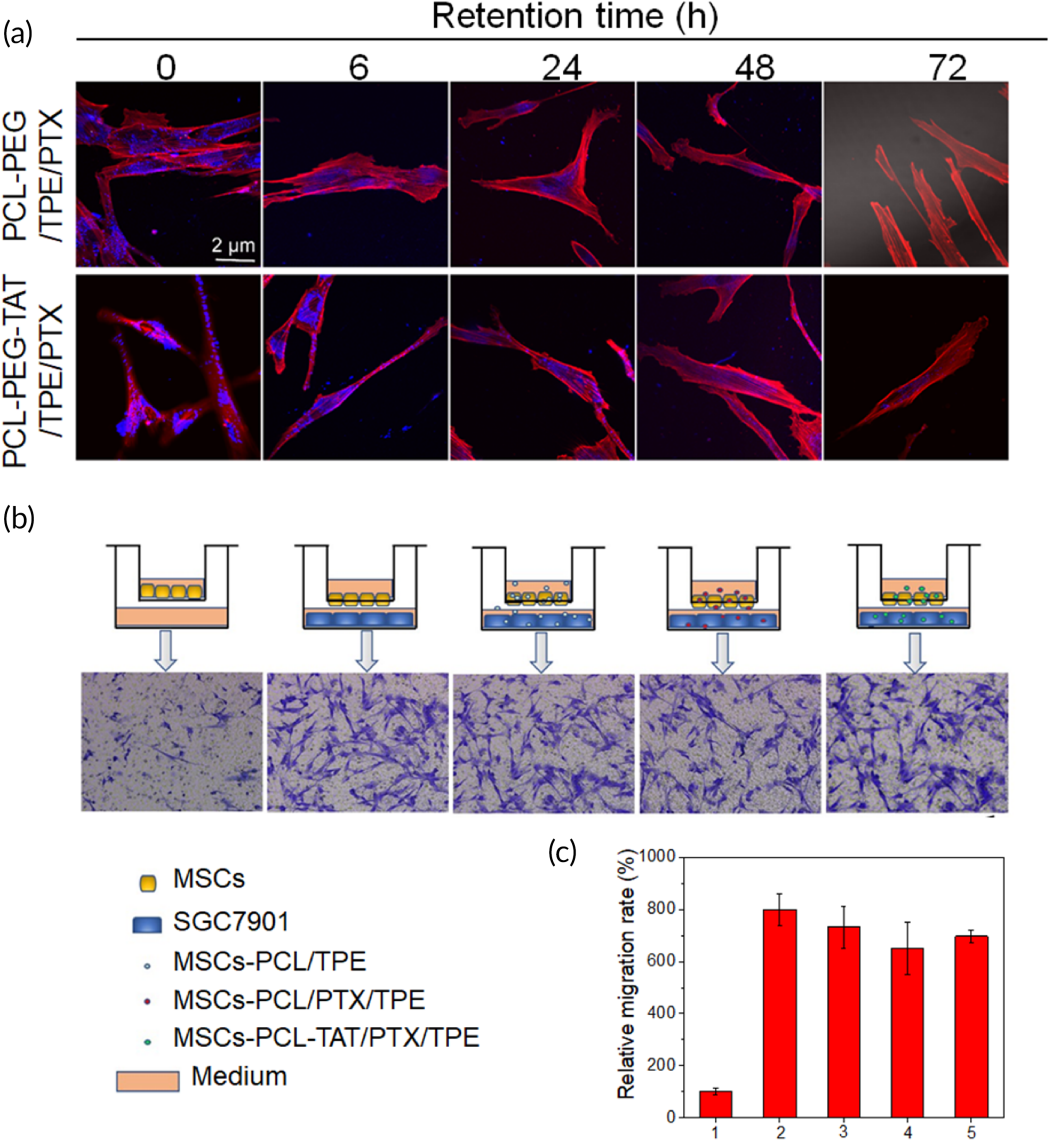
(a) Retention of PCL‐PEG/TPE/PTX with or without TAT modified in MSCs was observed via a confocal laser scanning microscopy (CLSM) at 0, 6, 24, 48, and 72 h. Red: Actin cytoskeletal morphology of MSCs stained with rhodamine phalloidin. Blue: Aggregation‐induced emission of TPE. (b) The images of MSCs migrated to SGC‐7901 cells with different treatments (from left to right: MSCs in the absence of target SGC‐7901 cells; MSCs incubated with nothing, PCL‐PEG/TPE, PCL‐PEG/PTX/TPE, and PCL‐PEG‐TAT/PTX/TPE in the presence of objective medium of SGC‐7901 cells) and stained with crystal violet, the transwell plate seeded with MSCs alone as the control. The pore size of transwell is 12 μm. (c) MSCs counts of transwell migration in different groups (MSCs in the absence of target SGC‐7901 cells (1), MSCs incubated with nothing (2), PCL‐PEG/TPE (3), PCL‐PEG/PTX/TPE (5), and PCL‐PEG‐TAT/PTX/TPE (6) when the objective medium of SGC‐7901 cells was in existence). MSC, mesenchymal stem cells; PCL‐PEG, polyethylenimine–poly(ε‐caprolactone); PTX, paclitaxel; TAT, transactivator of transcription; TPE, tetraphenylethylene

The drug retention of NPs in cells mainly depends on the efflux of cells and the dilution effects of cell division. We hypothesize that cell effluxion does not affect the retention time of NP drugs in cells. The MSCs were treated with PCL‐PEG/TPE/PTX and PCL‐PEG‐TAT/TPE/PTX for 6 h, and actin cytoskeletal staining was performed using rhodamine phalloidin to indicate morphology of MSCs. The contents in MSCs were observed through CLSM at set time intervals. As shown in Figure 4a, a strong signal belonging to TPE (blue fluorescence) was detected at 0 h, and the signal was still evident at 6 and 24 h, though the signal was weaker than the beginning. At 48 h, the signal weakened significantly. However, at 72 h, a faint fluorescence was observed in the MSCs treated with PCL‐PEG‐TAT/TPE/PTX. The retention effect in cells may be associated with the interaction of NPs and receptors on cells. The results suggested that TAT peptides could tightly bind to the membrane of MSCs, thus enhancing the uptake efficacy of MSCs.

### 3.7 Intracellular distribution of PCL‐PEG‐TAT/TPE

NP drug carriers are known for their high drug‐intake capacity. The intracellular distribution of PCL‐PEG‐TAT/TPE was further explored through CLSM. The Lyso‐Tracker‐Green stained the lysosomes fluorescent green, while TPE with AIE characteristics emitted blue fluorescence, indicating the distribution of PCL‐PEG‐TAT/TPE. Figure S5 shows the images of MCSs incubated with PCL‐PEG‐TAT/TPE for 0.5, 2, 4, and 6 h. PCL‐PEG‐TAT/TPE displayed a clear colocalization with Lyso‐Tracker, indicating the delivery of internalized NPs by MSCs to lysosomes. A weak blue fluorescence was observed at 30 min, indicating very little encapsulation of NPs by MSCs. The intensity of blue fluorescence increased over time, suggesting that PCL‐PEG‐TAT/TPE uptake by MSCs was time‐dependent. These data supported MSCs' great potential for NP drug intake.

### 3.8 In vitro migration assay

The tumor tropism of PCL‐PEG‐TAT/TPE/PTX loaded MSCs was assessed by in vitro migration assay. Only a few MSCs could pass through the membrane pores without an SGC‐7901 culture medium (Figure 4). The migrated MSCs from three random photos were counted (Figure 4b). In an SGC‐7901 medium, MSCs could easily migrate to the lower surface of the membrane (the migration increased 8.0 ± 0.61 times), suggesting the tumor‐tropic property of MSCs. When PCL‐PEG/TPE, PCL‐PEG/TPE/PTX, and PCL‐PEG‐TAT/TPE/PTX were separately internalized by MSCs, the migrated cell counts were 7.3 ± 0.81, 5.5 ± 1.01, and 6.2 ± 0.23 times of the control, respectively (Figure 4c). The results indicated that the migrating ability of MSCs was not affected significantly by the loading of the NP complex or NPs‐PTX. Compared to the bare MSCs, MSCs with PCL‐PEG‐TAT/TPE/PTX loading did not show a significant difference in migration. Preservation of MSC migration to tumor sites is a prerequisite when engineering MSCs with drug‐loaded NPs. The results suggested that MSCs could be employed as a vehicle for PCL‐PEG‐TAT/TPE/PTX transportation.

### 3.9 Cell apoptosis study

**FIGURE 5:**
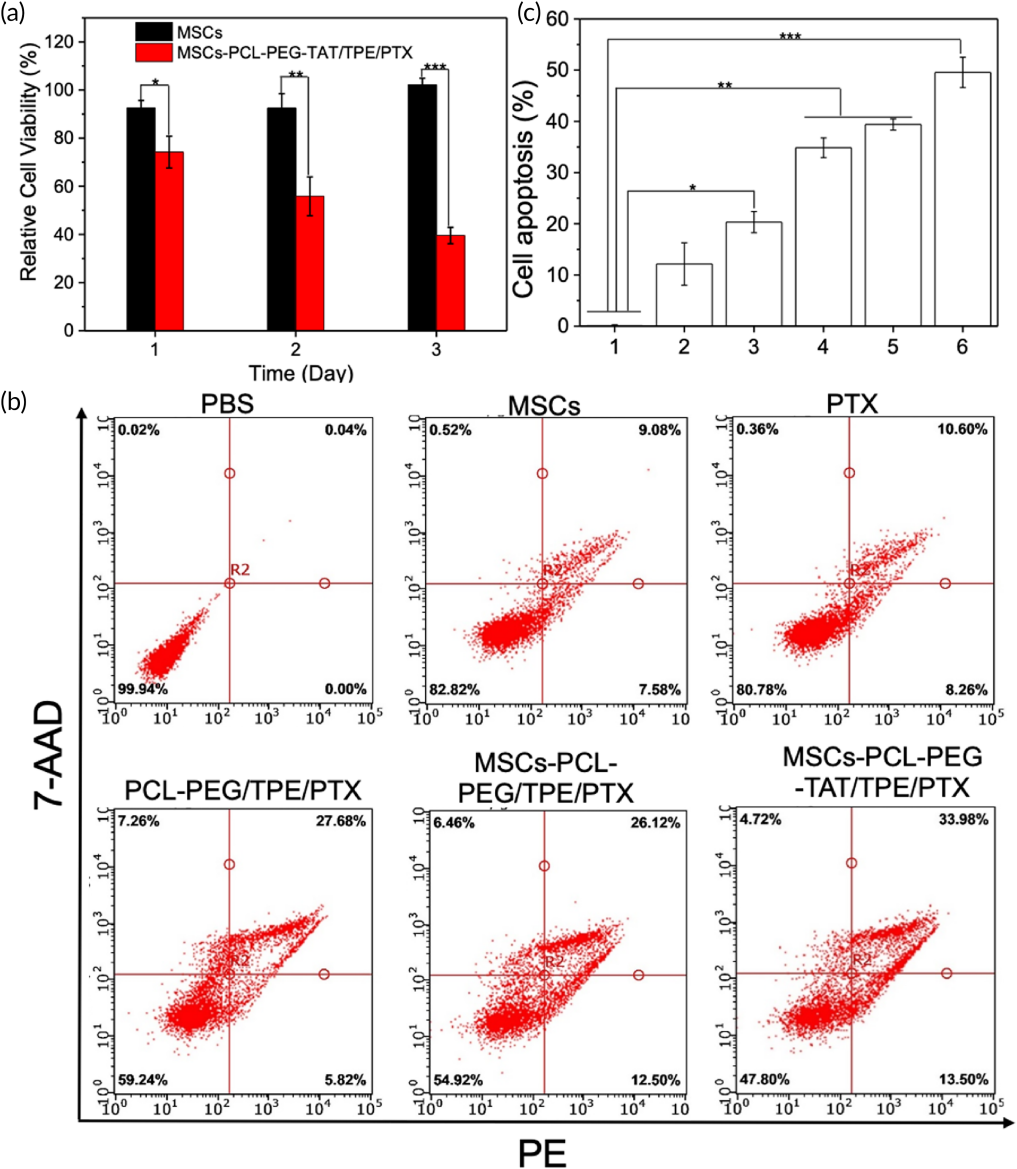
(a) Viability of SGC‐7901 cells cocultured with bare MSCs, and PCL‐PEG‐TAT/TPE/PTX loaded MSCs. Flow cytometric analysis (b) and apoptosis counts (c) of SGC‐7901 cells treated with PBS (1), MSCs (2), PTX (3), PCL‐PEG/TPE/PTX (4), MSC‐PCL‐PEG/TPE/PTX (5), and MSC‐PCL‐PEG‐TAT/TPE/PTX (6) (*p < 0.05, **p < 0.01, ***p < 0.001). MSC, mesenchymal stem cells; PBS, phosphate‐buffered saline; PCL‐PEG, polyethylenimine–poly(ε‐caprolactone); PTX, paclitaxel; TAT, transactivator of transcription; TPE, tetraphenylethylene

The classical anticancer agent, PTX, promotes microtubule assembly, inhibits cell proliferation and apoptosis induction.^36^ In this study, Annexin V‐PE/7‐AAD double staining was adopted to evaluate the apoptosis‐inducing capability of different agents on SGC‐7901 cells. PBS was used as the control. The flow cytometry scatter plot shows the apoptosis rate of SGC‐7901 cells to be 32% when treated with PCL‐PEG/TPE/PTX. The rate increased slightly to 38% when MSCs were utilized as an NP drug carrier, and the rate was further elevated to 47% when TAT was introduced due to its enhanced drug intake by the SGC‐7901 cells (Figure 5b,c, *p* < 0.05). These results indicated that MSCs have tropism to SGC‐7901 cells, and more NP drugs loaded by MSCs could enter the tumor cells. These results are in accordance with the in vitro antitumor effect.

### 3.10 In vivo distribution and tumor‐targeting ability

**FIGURE 6:**
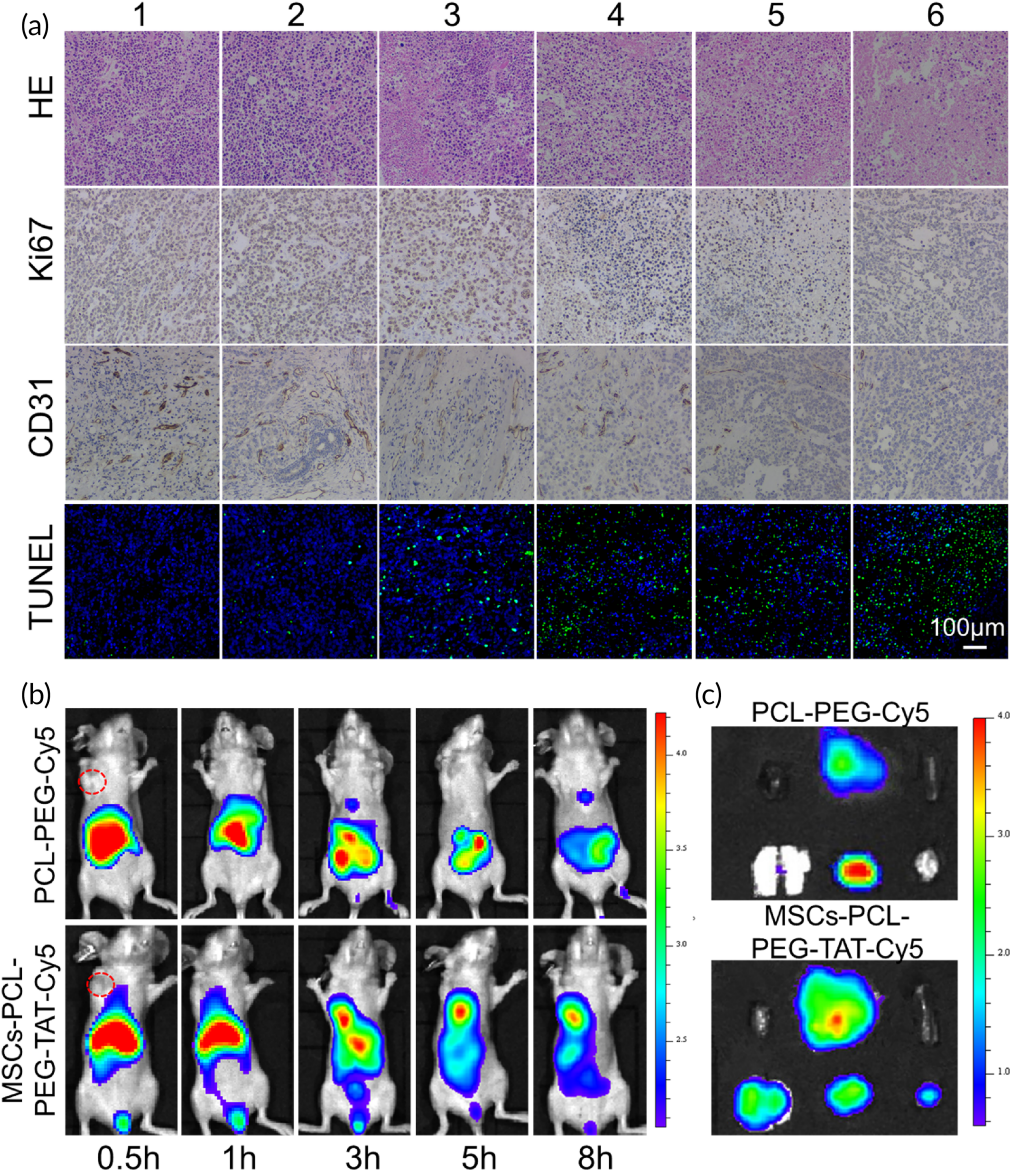
(a) H&E, TUNEL, and immunohistochemical staining of CD31 and Ki67. TUNEL‐positive are stained with green fluorescence. CD31‐positive and Ki67‐positive were stained with yellow–brown and brown color, respectively. (1: PBS, 2: MSCs, 3: free PTX, 4: PCL‐PEG/TPE/PTX, 5: MSCs‐PCL‐PEG/TPE/PTX, 6: MSCs‐PCL‐PEG‐TAT/TPE/PTX, *n* = 4). **(**b) Fluorescent image of xenograft tumor‐bearing nude mice after intravenous injections of PCL‐PEG‐Cy5 and MSCs‐PCL‐PEG‐TAT‐Cy5 at different times, *n* = 3. Tumors are indicated by red circles. (c) Fluorescent images of SGC‐7901 tumor sections and major organs at 8 h postinjection of PCL‐PEG‐Cy5 and MSCs‐PCL‐PEG‐TAT‐Cy5, respectively (from left to right: upper row: heart, liver and spleen, lower row: lung, kidney, and tumor). H&E, hematoxylin and eosin; MSC, mesenchymal stem cells; PBS, phosphate‐buffered saline; PCL‐PEG, polyethylenimine–poly(ε‐caprolactone); PTX, paclitaxel; TAT, transactivator of transcription; TPE, tetraphenylethylene; TUNEL, terminal deoxynucleotidyl transferase dUTP nick end labeling

In vivo biodistribution and tumor‐targeting ability were further investigated by intravenously injecting PCL‐PEG‐Cy5 and MSCs‐PCL‐PEG‐TAT‐Cy5 into SGC‐7901 xenograft tumor‐bearing mice (2 mg/kg, *n* = 3) respectively. It was observed, for MSCs carrying NPs, that tumor accumulation occurred at 60‐min postinjection and peaked at 3‐h postinjection, while the fluorescence intensity gradually decreased over time, with accumulation evident in tumor tissues (Figure 6b). In contrast, when NPs were injected without MSCs, no significant fluorescence signal was observed in the tumor sites over time. Figure 6a shows the biodistribution of the tumor and major organs harvested at 8‐h postinjection. Accumulation of PCL‐PEG‐Cy5 was only observed in the kidneys, and no accumulation was observed in the tumor tissues. Furthermore, the MSCs‐PCL‐PEG‐TAT‐Cy5 displayed a higher tumor accumulation than the NPs without MSCs due to tumor tropism of MSCs. In addition, consistent with previous studies,^37^ we also observed that the drug‐loaded NPs‐primed MSCs remained in the lungs due to mechanical entrapment within the lung capillary beds; however, the rapid entrapment disappeared gradually, followed by the redistribution of MSCs in the tumor sites. The results distinctly suggest that MSCs carrying NPs possess tumor‐targeting ability.

### 3.11 In vivo toxicity analysis

**FIGURE 7:**
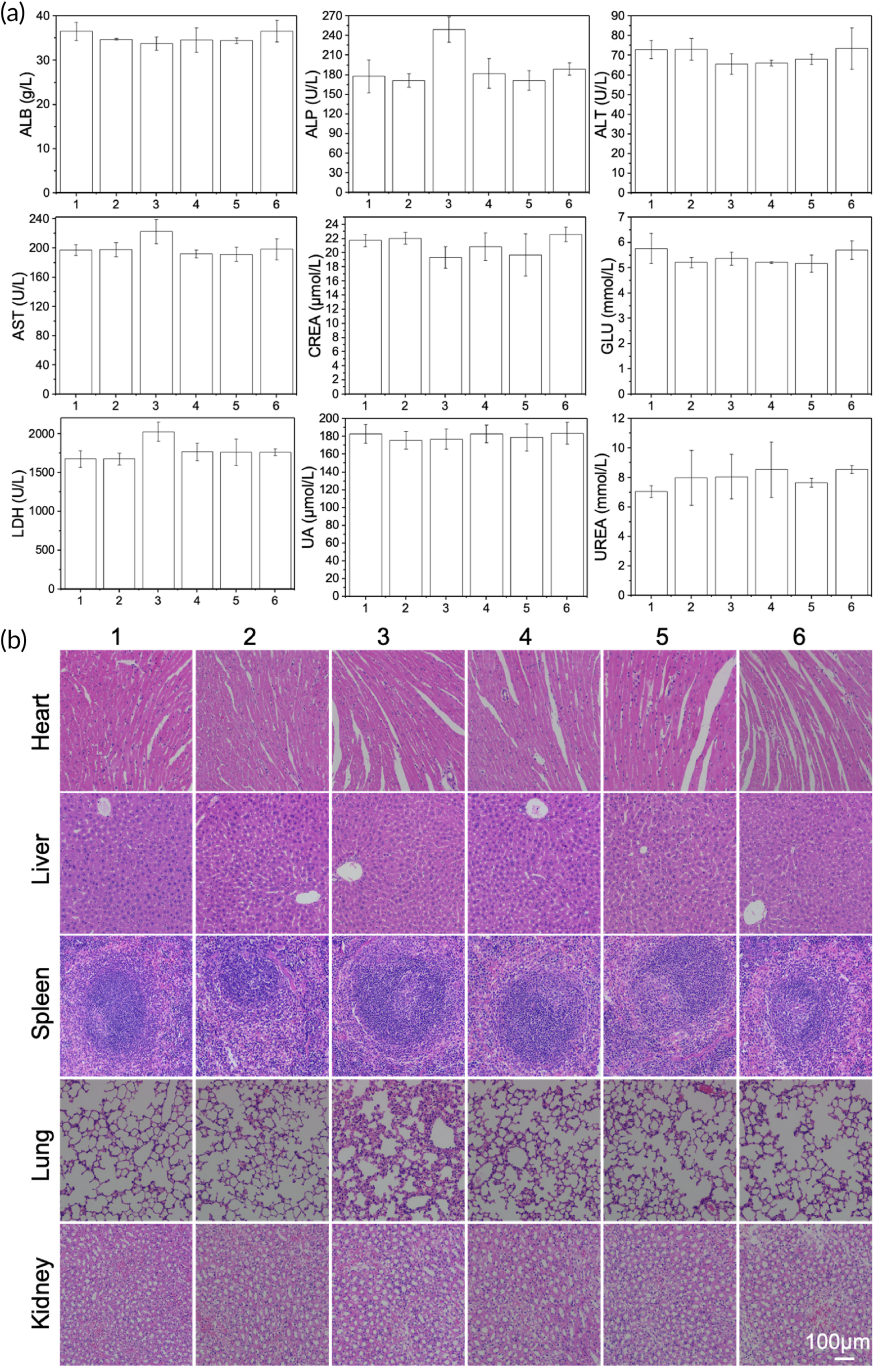
Assessment of serum levels of ALB, ALP, ALT, AST, LDH, GLU, CREA, UA, and UREA (a) and histological sections of the major organs (b) of mice after treated with PBS (1), MSCs (2), free PTX (3), PCL‐PEG/TPE/PTX (4), MSCs‐PCL‐PEG/TPE/PTX (5), and MSCs‐PCL‐PEG‐TAT /TPE/PTX (6) for 21 days. Values represent mean ± standard deviation (*SD*), *n* = 4. H&E staining; magnification: ×100. ALB, albumin; ALP, alkaline phosphatase; ALT, alanine aminotransferase; AST, aspartate aminotransferase; CREA, creatinine; GLU, glucose; LDH, lactate dehydrogenase; MSC, mesenchymal stem cells; PBS, phosphate‐buffered saline; PCL‐PEG, polyethylenimine–poly(ε‐caprolactone); PTX, paclitaxel; TAT, transactivator of transcription; TPE, tetraphenylethylene; UA, uric acid; UREA, urea

The study evaluated the potential side effects of different formulations in vivo. After 21 days of treatment, hematological parameters including ALT, AST, LDH, ALB, ALP, GLU, CREA, UA, and UREA were similar except the group treated with free PTX had a significant elevation of AST, ALP, and LDH levels (Figure 7a), suggesting that the constructed NP drugs and MSCs carrying NP drugs possessed good biosafety. Furthermore, no noticeable organ lesions (heart, liver, spleen, lungs, and kidneys) were observed by histological analysis (Figure 7b). The histological data demonstrated good biocompatibility of the MSCs‐PCL‐PEG‐TAT/TPE/PTX as a drug‐delivery platform.

### 3.12 In vivo antitumor efficacy

**FIGURE 8:**
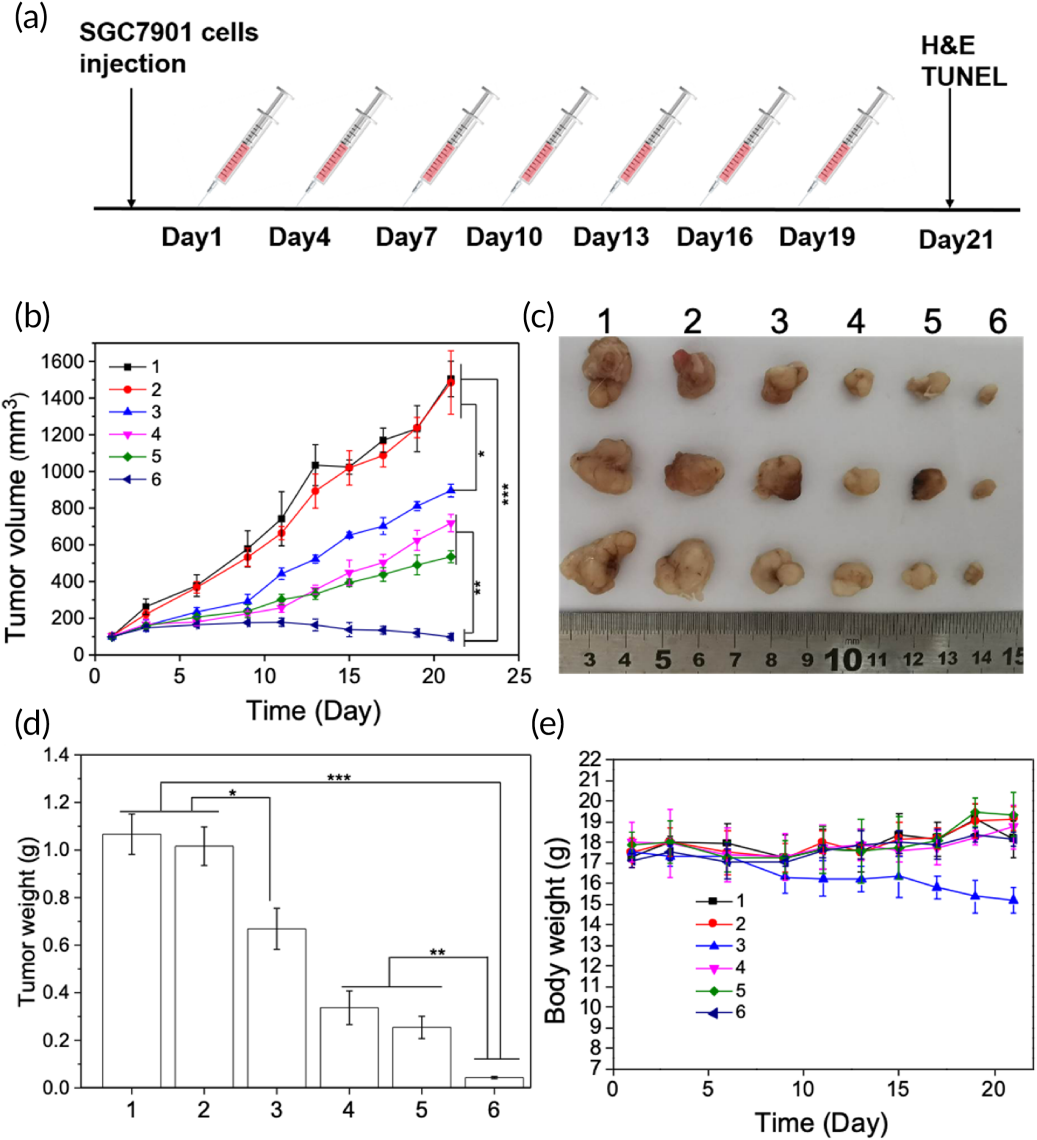
In vivo antitumor ability of different agents administrated intravenously. (a) Illustration of the injection intervals. (b) Tumor volume of the SGC‐7901 xenograft tumor‐bearing mice after treatment with PBS, MSCs, free PTX, PCL‐PEG/TPE/PTX, MSCs‐PCL‐PEG/TPE/PTX, and MSCs‐PCL‐PEG‐TAT/TPE/PTX. (c) Harvested SGC‐7901 gastric tumors and (d) average tumor weight of each group (*p < 0.05, **p < 0.01, ***p < 0.001). (e) Mean body weight of each group during the 21‐day treatment period (1: PBS, 2: MSCs, 3: free PTX, 4: PCL‐PEG/TPE/PTX, 5: MSCs‐PCL‐PEG/TPE/PTX, 6: MSCs‐PCL‐PEG‐TAT/TPE/PTX, *n* = 4). MSC, mesenchymal stem cells; PBS, phosphate‐buffered saline; PCL‐PEG, polyethylenimine–poly(ε‐caprolactone); PTX, paclitaxel; TAT, transactivator of transcription; TPE, tetraphenylethylene

In vivo therapeutic efficacy was evaluated by intravenously injecting different formulations into SGC‐7901 tumor‐bearing nude mice (*n* = 4, once every 3 days; Figure 8a). Antitumor activity and tolerability were determined by the measurement of the alterations in the tumor size and bodyweight of the mice (Figure 8). The tumor volume of the mice treated with PBS and bare MSCs rapidly increased to about 1500 mm^3^ within 21 days, indicating no tumor inhibition by both formulations. In contrast, the PCL‐PEG/TPE/PTX showed a significant influence on the inhibition of tumor growth, and the tumor volume was half of that treated with PBS and bare MSCs. PTX without NPs exhibited inferior therapeutic efficacy than NPs‐PTX. The PCL‐PEG/TPE/PTX loaded by MSCs showed better tumor inhibition than PCL‐PEG/TPE/PTX, and the TAT‐peptide introduction further enhanced the therapeutic effect (Figure 8b,d, *p* < 0.05). Figure 8c displays the excised tumor tissue showing the remarkable antitumor activity of NP drugs and MSCs‐NP drugs. The side effect was evaluated by the mice's weight changes (Figure 8e). A distinct body weight loss was caused by free PTX treatment. While the administration of the NP drugs, MSCs‐NP drugs, and PBS formulations shows no obvious effect on the mice body weight, suggesting that MSCs and NPs possess high biocompatibility and can reduce the off‐target effect by localizing the drug at tumor sites with a controllable release.

### 3.13 In vivo antitumor mechanism study

After 21 days of treatment, the histology of tumor tissues was analyzed by the H&E staining and TUNEL staining. Blue represents nuclei of the hematoxylin‐labeled cells, while red indicates the eosin‐labeled cytoplasm in H&E. The H&E analysis results further confirmed that the MSCs‐PCL‐PEG‐TAT/TPE/PTX was the most effective in inducing cell necrosis, with the most deformed nuclei and larger intercellular spaces. The TUNEL staining exhibited varying degrees of apoptosis induced by different NPs, while the MSCs‐PCL‐PEG‐TAT/TPE/PTX exhibited the most extensive DNA‐damage effect. In addition, immunostaining Ki67 and CD31 was performed to investigate the antiproliferation and antiangiogenesis effects of MSCs‐PCL‐PEG‐TAT/TPE/PTX. The cells expressing Ki67 and CD31 were stained light brown and dark brown, respectively. The tumor tissues of the MSCs‐PCL‐PEG‐TAT/TPE/PTX‐treated mice exhibited a markedly lower count of Ki‐67 positive and CD31 positive cells than PBS or other groups (Figure 6a). The findings demonstrated that MSCs‐PCL‐PEG‐TAT/TPE/PTX could induce extensive apoptosis and antiangiogenesis.

## 4 CONCLUSION

We successfully developed an MSC‐based DDS with good biocompatibility and significant suppression of the tumor cell growth. The synthesized PCL‐PEG‐TAT, with a size of around 118 nm and regular sphere shape in an aqueous solution, was used for carrying the anticancer drug, PTX. In this study, PTX loaded onto NPs‐TAT significantly reduced the removal of NPs by the RES, providing a longer NPs retention time within the tumor. In addition, NPs decorated with TAT peptide enhanced the cellular uptake. Finally, MSCs as cell vehicles could target tumors efficiently. The results of in vitro and in vivo demonstrated the feasibility of MSCs as the carriers of NPs‐TAT/PTX; this cellular‐based drug NP platform could be applied for the on‐demand delivery of other therapeutic drugs, target treatments, and diagnosis of various malignancies.

## AUTHOR CONTRIBUTIONS

**Senlin Zhu:** Conceptualization (lead); funding acquisition (lead); project administration (lead); supervision (lead); writing – review and editing (lead). **Sushan Ouyang:** Investigation (equal); methodology (equal); writing – original draft (lead). **Yi Zhang:** Investigation (equal); methodology (equal). **Sheng Yao:** Investigation (equal); methodology (equal). **Longbao Feng:** Methodology (supporting). **Ping Li:** Investigation (supporting).

## CONFLICT OF INTERESTS

The authors declare no conflict of interests.

## Supporting information


**Figure S1** ^1^H‐NMR spectrum of PCL‐PEG‐NH_2_, TPE, and PTX.
**Figure S2.** FT‐IR spectrum of PCL‐PEG/TPE/PTX, PCL‐PEG‐NH_2_, TPE, and PTX. Characteristic peaks indicated with dash lines and arrows.
**Figure S3.** Fluorescence intensity with different concentrations at 442 nm.
**Figure S4.** The live/dead staining of SGC‐7901 cells after 3‐day coculture with bare MSCs and PCL‐PEG‐TAT/TPE/PTX‐primed MSCs.
**Figure S5.** Intracellular fluorescence distribution in MSCs after incubation with PCL‐PEG‐TAT/TPE for 0.5, 2, 4, and 6 h. Lysosomes were stained with Lyso‐Tracker‐Green. TPE emitted blue fluorescence. Endocytosis analysis was done through CLSM.
**Figure S6.** (A) Retention of PCL‐PEG/TPE/PTX with or without TAT modified in MSCs was observed via a confocal laser scanning microscope (CLSM) at 0, 6, 24, 48, and 72 h. Red: actin cytoskeletal morphology of MSCs stained with rhodamine phalloidin. Blue: aggregation‐induced emission of TPE. (*P < 0.05, **P < 0.01, ***P < 0.001).
**Figure S7.** (A) H&E, TUNEL, and immunohistochemical CD31 and Ki67 staining. TUNEL‐positive are stained with green fluorescence. CD31‐postive and Ki67‐positive were stained with yellow–brown and brown color, respectively. (1: PBS, 2: MSCs, 3: free PTX, 4: PCL‐PEG/TPE/PTX, 5: MSCs‐PCL‐PEG/TPE/PTX, 6: MSCs‐PCL‐PEG‐TAT/TPE/PTX, *n* = 4). (*P < 0.05, **P < 0.01, ***P < 0.001).

## Data Availability

Data openly available in a public repository that issues datasets with DOIs.
