# Supplementary material for: The efficiency of MSC‐based targeted AIE nanoparticles for gastric cancer diagnosis and treatment: An experimental study
Source: Bioeng Transl Med. 2021 Dec 24;7(2):e10278. doi: 10.1002/btm2.10278 (PMC9115694; doi:10.1002/btm2.10278)
Supplement: Supplementary file 1 — Figure S1 1H‐NMR spectrum of PCL‐PEG‐NH2, TPE, and PTX. Figure S2. FT‐IR spectrum of PCL‐PEG/TPE/PTX, PCL‐PEG‐NH2, TPE, and PTX. Characteristic peaks indicated with dash lines and arrows. Figure S3. Fluorescence intensity with different concentrations at 442 nm. Figure S4. The live/dead staining of SGC‐7901 cells after 3‐day coculture with bare MSCs and PCL‐PEG‐TAT/TPE/PTX‐primed MSCs. Figure S5. Intracellular fluorescence distribution in MSCs after incubation with PCL‐PEG‐TAT/TPE for 0.5, 2, 4, and 6 h. Lysosomes were stained with Lyso‐Tracker‐Green. TPE emitted blue fluorescence. Endocytosis analysis was done through CLSM. Figure S6. (A) Retention of PCL‐PEG/TPE/PTX with or without TAT modified in MSCs was observed via a confocal laser scanning microscope (CLSM) at 0, 6, 24, 48, and 72 h. Red: actin cytoskeletal morphology of MSCs stained with rhodamine phalloidin. Blue: aggregation‐induced emission of TPE. (*P < 0.05, **P < 0.01, ***P < 0.001). Figure S7. (A) H&E, TUNEL, and immunohistochemical CD31 and Ki67 staining. TUNEL‐positive are stained with green fluorescence. CD31‐postive and Ki67‐positive were stained with yellow–brown and brown color, respectively. (1: PBS, 2: MSCs, 3: free PTX, 4: PCL‐PEG/TPE/PTX, 5: MSCs‐PCL‐PEG/TPE/PTX, 6: MSCs‐PCL‐PEG‐TAT/TPE/PTX, n = 4). (*P < 0.05, **P < 0.01, ***P < 0.001). [file BTM2-7-e10278-s001.docx]

**Supporting Information**

**The Efficiency of MSC-Based Targeted AIE Nanoparticles for Gastric Cancer Diagnosis and Treatment: An Experimental Study**

Sushan Ouyang ^# 1^, Yi Zhang ^# 2^, Sheng Yao ^# 1,3^, Longbao Feng ^4^, Ping Li^1^, Senlin Zhu^1*^

1 *Department of Gastroenterology and Hepatology, The First Affiliated Hospital of Sun Yat-sen University, Guangzhou, 510080, China*

2 *Department of Hepatobiliary Surgery, The Third Affiliated Hospital of Sun Yat-sen University, Guangzhou, 510630, China*

3 *Department of Gastroenterology, The Second Affiliated Hospital of Zhejiang University School of Medicine, Hangzhou, 310009, China*

4 *Beogene Biotech (Guangzhou) Co., Ltd., Guangzhou, 510663, China*

# These authors contributed equally to this work.

* Correspondence to: Senlin Zhu; E-mail: [zhusl@mail.sysu.edu.cn](mailto:zhusl@mail.sysu.edu.cn).

**Materials and Methods**

MSC culture

MSCs, derived from bone marrow of SD rats, were supplied by Guangzhou Beogene Biotech Co., Ltd., (Guangzhou, China). The second passage number (P2) was received frozen in liquid nitrogen and preseved at –120 °C. Ahead of using it, MSCs were thawed and grown in L-DMEM with the addition of FBS (10%, Gibco) and penicillin-streptomycin (1%, Thermo Fisher) in a 25 cm^2^ flask at 37 °C. When 80% confluence was reached, MSCs were subcultured at a ratio of 1:3 and expanded to the passages 3–6 for use.

**Figure captions**

**Figure S1.** ^1^H-NMR spectrum of PCL-PEG-NH_2_, TPE, and PTX.

**Figure S2.** FT-IR spectrum of PCL-PEG/TPE/PTX, PCL-PEG-NH_2_, TPE, and PTX. Characteristic peaks indicated with dash lines and arrows.

**Figure S3.** Fluorescence intensity with different concentrations at 442 nm.

**Figure S4.** The live/dead staining of SGC-7901 cells after 3-day coculture with bare MSCs and PCL-PEG-TAT/TPE/PTX-primed MSCs.

**Figure S5.** Intracellular fluorescence distribution in MSCs after incubation with PCL-PEG-TAT/TPE for 0.5, 2, 4, and 6 h. Lysosomes were stained with Lyso-Tracker-Green. TPE emitted blue fluorescence. Endocytosis analysis was done through CLSM.

**Figure S6.** (A) Retention of PCL-PEG/TPE/PTX with or without TAT modified in MSCs was observed via a confocal laser scanning microscope (CLSM) at 0, 6, 24, 48, and 72 h. Red: actin cytoskeletal morphology of MSCs stained with rhodamine-phalloidin. Blue: aggregation-induced emission of TPE. (**P* < 0.05, ***P* < 0.01, ****P* < 0.001).

**Figure S7.** (A) H&E, TUNEL, and immunohistochemical CD31 and Ki67 staining. TUNEL-positive are stained with green fluorescence. CD31-postive and Ki67-positive were stained with yellow-brown and brown color, respectively. (1: PBS, 2: MSCs, 3: free PTX, 4: PCL-PEG/TPE/PTX, 5: MSCs-PCL-PEG/TPE/PTX, 6: MSCs-PCL-PEG-TAT/TPE/PTX, *n* = 4). (**P* < 0.05, ***P* < 0.01, ****P* < 0.001).


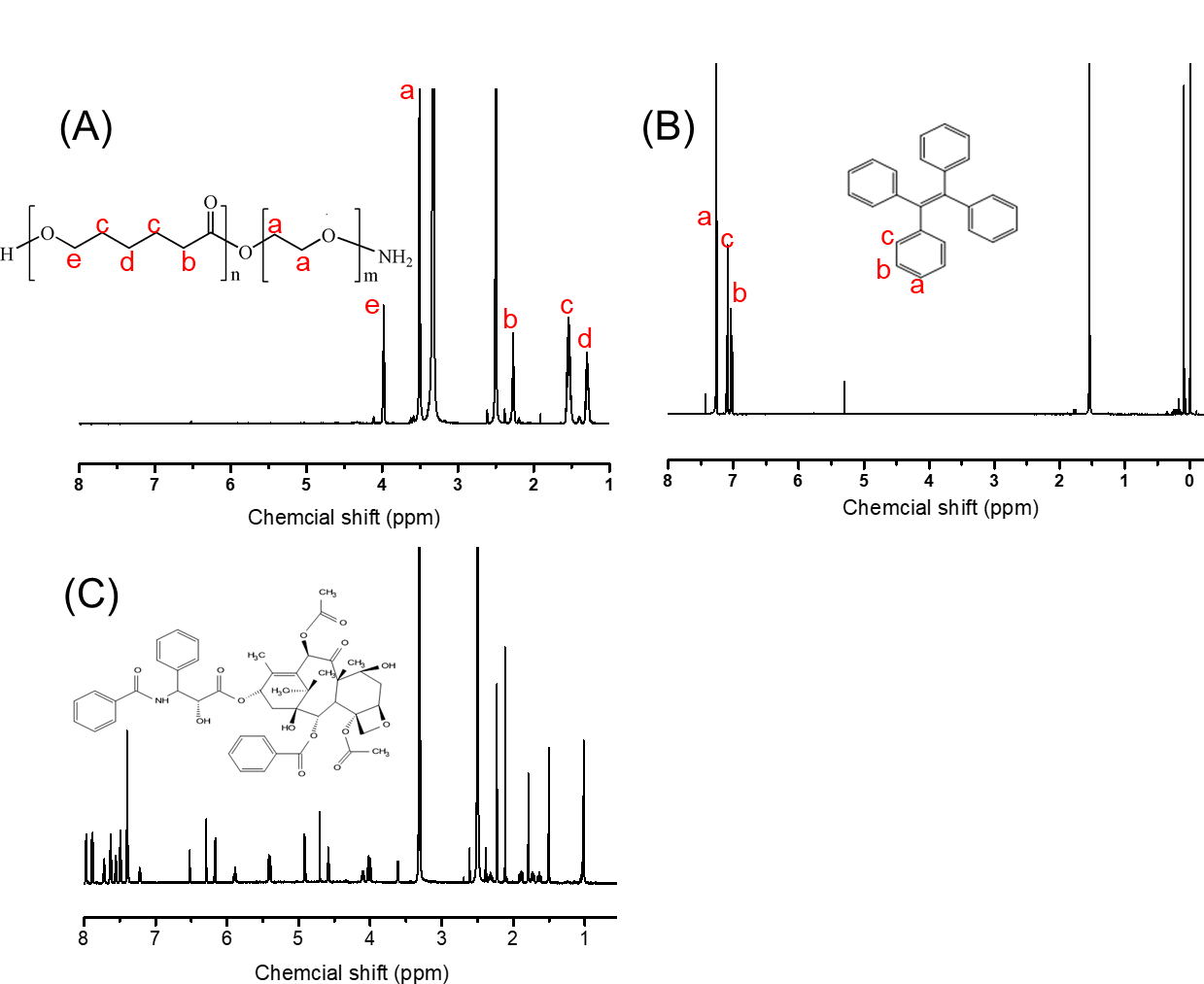


**Figure S1.**


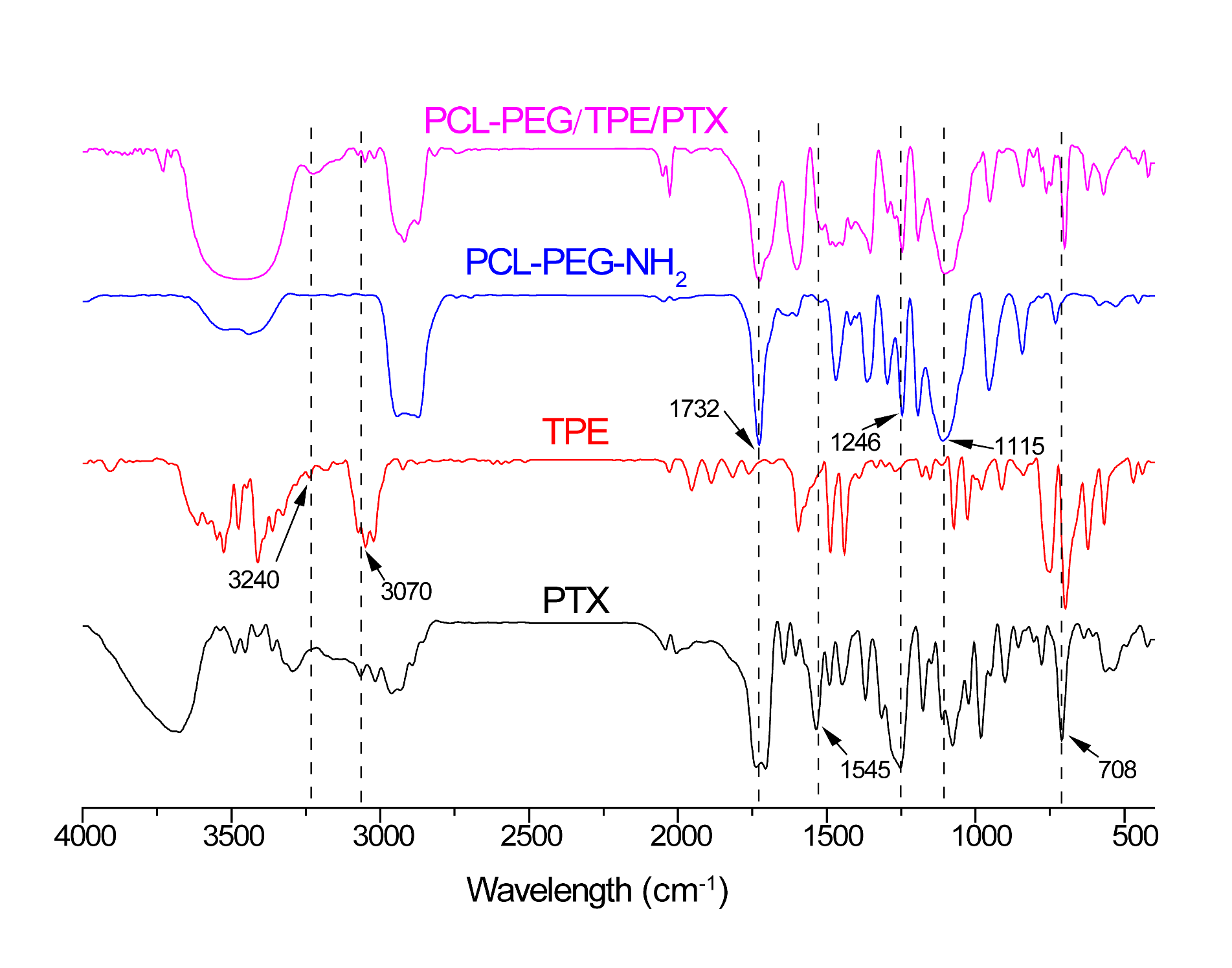


**Figure S2.**





**Figure S3.**


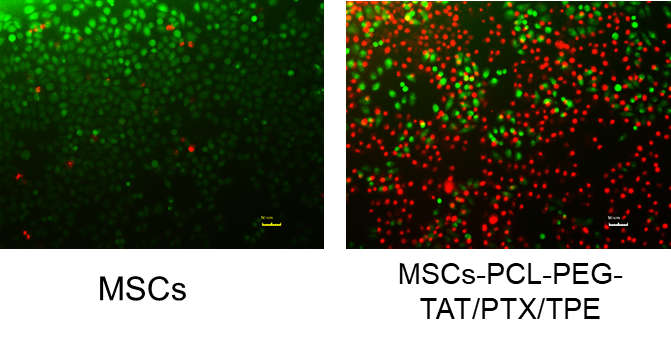


**Figure S4.**


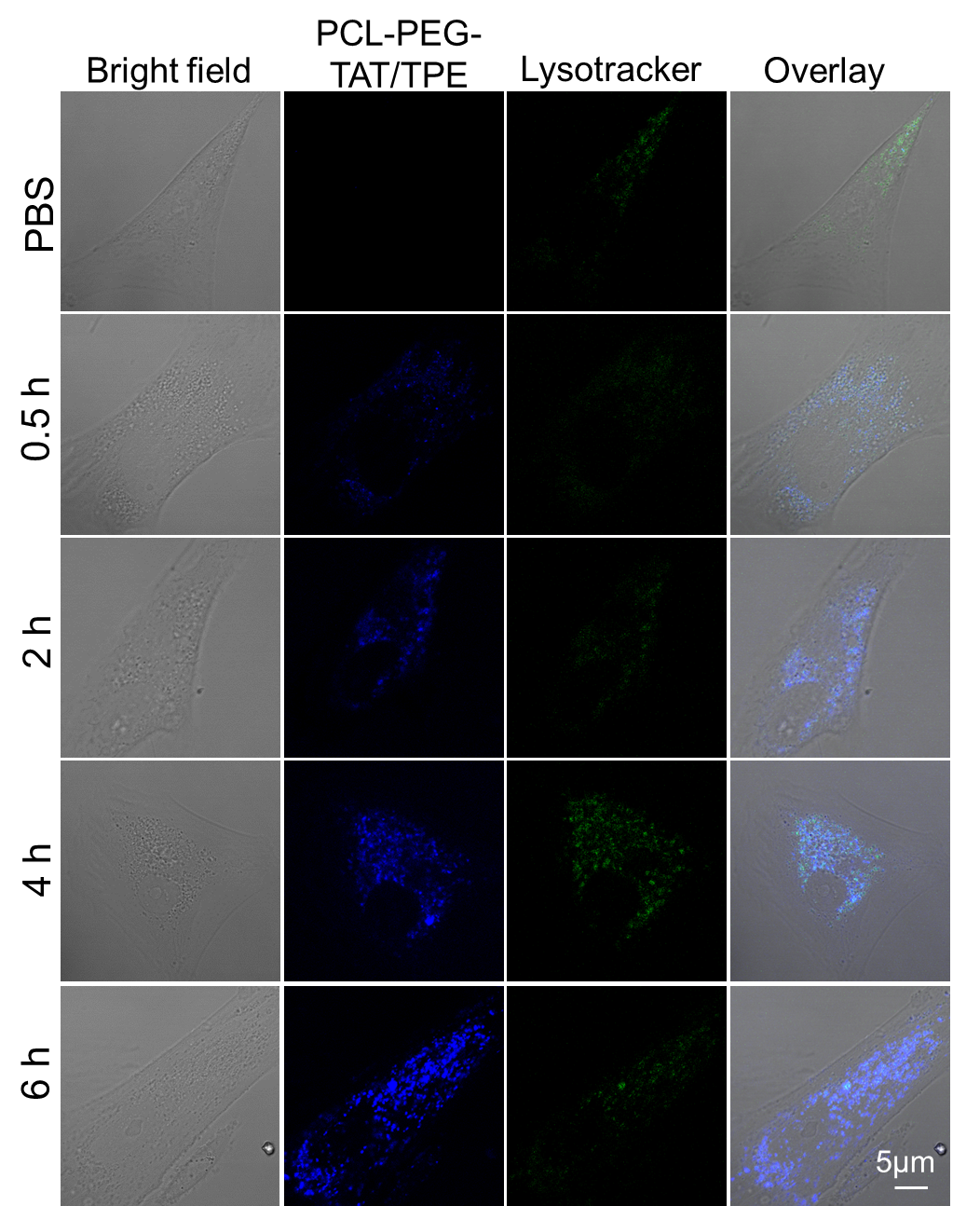


**Figure S5.**

**Figure S6.**

**Figure S7.**
